# Optimized plasma-assisted bi-layer photoresist fabrication protocol for high resolution microfabrication of thin-film metal electrodes on porous polymer membranes

**DOI:** 10.1016/j.mex.2019.10.038

**Published:** 2019-11-09

**Authors:** Patrick Schuller, Mario Rothbauer, Christoph Eilenberger, Sebastian R.A. Kratz, Gregor Höll, Philipp Taus, Markus Schinnerl, Jakob Genser, Peter Ertl, Heinz Wanzenboeck

**Affiliations:** a Institute of Applied Synthetic Chemistry and Institute of Chemical Technologies and Analytics, Vienna University of Technology, Vienna, Austria; b Institute of Solid-State Electronics, Vienna University of Technology, Vienna, Austria

**Keywords:** Bi-layer photoresist lift-off, Micro fabrication, Track-etched membranes, Metal thin-film electrodes, Bi-layer lift-off

## Abstract

Structured metal thin-film electrodes are heavily used in electrochemical assays to detect a range of analytes including toxins, biomarkers, biological contaminants and cell cultures using amperometric, voltammetric and impedance-based (bio)sensing strategies as well as separation techniques such as dielectrophoresis. Over the last decade, thin-film electrodes have been fabricated onto various durable and flexible substrates including glass, silicon and polymers. However, the combination of thin-film technology with porous polymeric substrates frequently used for biochips often results in limited resolution and poor adhesion of the metal thin-film, thus severely restricting reproducible fabrication and reliable application in e.g. organ-on-a-chip systems. To overcome common problems associated with micro-structured electrode manufacturing on porous substrates, we have optimized a bi-layer lift-off method for the fabrication of thin-film electrodes on commercial porous polyester membranes using a combination of LOR3A with AZ5214E photoresists. To demonstrate practical application of our porous electrode membranes for trans-epithelial electrical resistance measurements a tetrapolar biosensing set-up was used to eliminate the artificial resistance of the porous polymer membrane from the electrochemical recordings. Furthermore, barrier resistance of Bewo trophoblast epithelial cells was compared to a standard Transwell assay readout using a EVOM2 volt-ohm meter.

• Bi-layer photo resist lift-off yields resolution down to 2.5 μm.
• Argon Plasma-assisted lift-off results in improved adhesion of gold thin films and eliminates the need for chromium adhesion layers.
• Membrane electrodes can be used for elimination of the porous membrane resistance during tetra-polar epithelial resistance measurements.

## Graphical abstract

**Figure:**
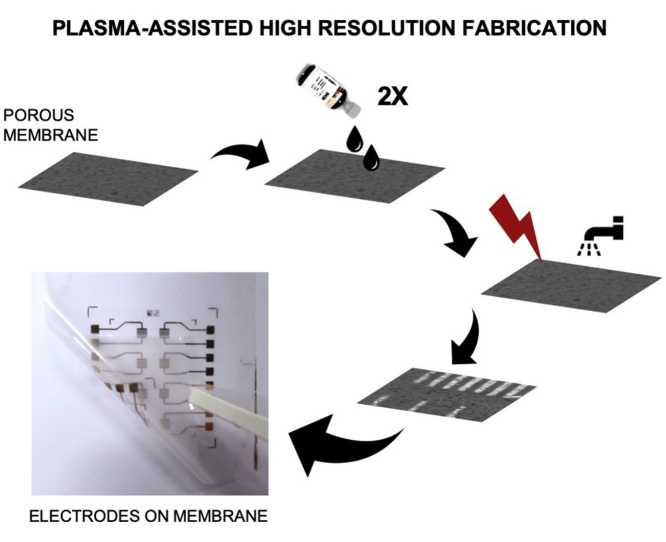

Specification Table

**Table:** 

Subject area:	Microfabrication
More specific subject area:	Sensor microfabrication
Method name:	Bi-layer photoresist lift-off
Name and reference of original method:	[1,2]
Resource availability:	http://microchem.com/Prod-PMGI_LOR.htmhttps://www.microchemicals.com/products/photoresists/az_5214_e.html

## Method details

### Preparation of membranes with integrated electrodes

#### Materials

• Clean room facility
• Plastic consumables: cell culture dishes and flasks, serological pipettes, syringes and centrifuge tubes.
• Hot plate
• Spin coater
• O_2_/Ar plasma asher or Reactive Ion Etching system
• Sputter deposition system/ Evaporation system
• syringe filters (22 μm)
• Scalpel
• UV chamber
• Porous track-etched PET Membrane (e.g.: ipCELLCULTURE™ Track Etched Membrane POLYESTER (PET) – Product Reference: 2000M12/580M303/R3, it4ip S.A., Belgium)
• LOR3A resist
• AZ5214E resist (can be substituted with other negative resists)
• AZ726MIF developer
• low molecular weight PVA (13000–23000 Da).
• deionized H_2_O (diH_2_O)
• Acetone
• Isopropyl alcohol
• *N*-methyl pyrrolidone or *N*-Ethyl pyrrolidone

#### Procedure

**Fig. 1:**
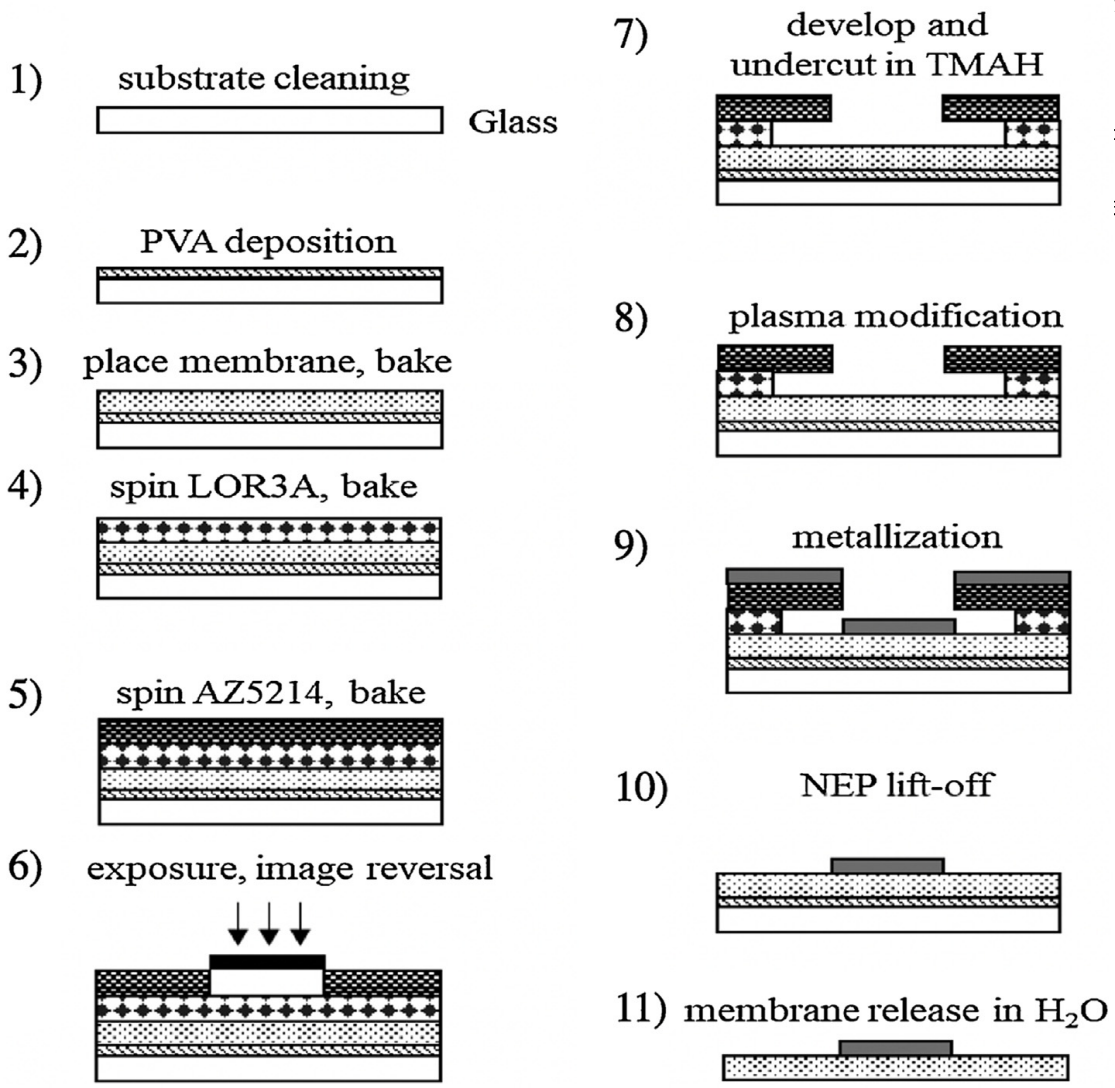
Overview of the optimized protocol for plasma-assisted high resolution manufacturing of thin-film electrodes on porous polymer membranes.

*Note:* Higher molecular weight PVA is more viscous and might hinder the membrane release after fabrication.

*Note:* Try to avoid wrinkles (it helps to bend the membrane a little) – once the membrane has been in contact with PVA it shouldn’t be moved anymore!

*Note*: Place the membrane onto the carrier while the PVA is still wet!

The process flow is illustrated in Fig. 1.

1 Dissolve 4 g of PVA in 100 ml deionized H_2_O (diH_2_O).
2 Stir at 70 °C (covered with aluminum foil to prevent evaporation of water) until the PVA is fully dissolved.
3 Once the PVA is dissolved, filter the solution through a syringe filter (22 μm) to remove particles.
4 Wait until PVA is at room temperature.
5 Clean the glass carrier substrate (Schott D263T eco) using Acetone and Isopropyl alcohol.
6 Place it on a hot plate and set it to 100 °C to evaporate any remaining solvent.
7 Treat glass substrate with O_2_ plasma (300 W; 0.7 Torr; 45 s) to allow easier spreading of the PVA release layer.
8 Transfer the plasma treated glass substrates to a spin coater and spread PVA using a transfer pipette or syringe and then spin at 800 rpm for 30 s.
9 After spin coating of the PVA release layer, pre-cut PET membrane piece (slightly larger than the carrier substrate) are carefully place it on the carrier substrate.

10 In order to dry the PVA on the substrates with the membrane attached, place it on a hotplate and ramp temperature to 150 °C (the LOR3A resist needs to be baked at this temperature).

*Note:* If no hotplate with a ramping function is available, or the ramping is too time consuming – the samples can be also baked gradually using hotplate set to 70 °C, 100 °C, 120° and 150 °C for 180 s each.

*Note*: If the samples are baked too fast the evaporating water will cause wrinkles on the membrane.

11 After dehydration cool the samples to room temperature and cut membrane pieces that are overlapping the carrier substrate using a scalpel
12 Spin coat LOR3A resist at 1000 rpm for 30 s and then soft bake at 150 °C for 180 s.

*Note:* The temperature should be ramped (or as mentioned above baked gradually).

13 Once the LOR3A has been soft baked, AZ5214E resist (or a simple negative resist) is spin coated at 3000 rpm for 30 s and then soft baked at 100 °C for 30 s.
14 Using a photo mask, transfer the desired electrode geometry to the sample by UV light (365 nm) exposure with a dose of 40 mJ/cm².
15 After exposure, bake the sample at 120 °C for 70 s and then flood expose (without photo mask) with a dose of 240 mJ/cm².
16 Develop sample in AZ726MIF (TMAH based developer) for 120 s and rinse with diH_2_O.

*Note:* usually AZ5214E needs to be developed for 60 s – but TMAH dissolves LOR3A and allows for an undercut of the actual photo resist

17 Dry the samples (e.g. with Nitrogen spray gun, overnight in a desiccator).
18 Before depositing the metal layer, subject samples to an Argon plasma (50 W RF; 10 sccm Ar; 60 s), thereby modifying the parts of the membrane not covered by photoresist. (this can be done with a plasma asher or a Reactive Ion Etching System (RIE), we used a RIE because the power can be adjusted more precisely)
19 Deposit a gold layer of approximately 80 nm by sputtering (25 W, 2 × 60 s sputter duration, base pressure: 2*10^-5^ mbar, working pressure 8*10^-3^ mbar) or evaporation.

*Note:* The sputter power should not exceed 25 W, otherwise the membrane might overheat, or the metal might crack or spall during lift-off because of strain/tensile forces.

*Note:* Deposition can be also done with an evaporation system

*Note:* In case different metals are to be used, plasma treatment with a different gas species might improve adhesion (we found that O_2_ plasma improved adhesion of Chromium)

*Note:* Protocols for the fabrication of membrane integrated electrodes have been reported before [1], these protocols didn’t include a plasma treatment prior to metal deposition – we found that this step improves adhesion of Chromium and Gold layers to the cell culture treated PET membranes supplied by it4ip, Belgium.

*Note:* In case other PET membranes from other suppliers are used, the plasma parameters might change.

20 After sputtering, soak the samples in *N*-methyl pyrrolidone or *N*-Ethyl pyrrolidone for 10 min and sonicate at low power to remove the photo resist and non-patterned gold.
21 Release the membrane by soaking the sample in diH_2_O for 1 min and then carefully pull it off with tweezers.
22 Using the process, gold electrodes can be deposited on porous membranes achieving a resolution down to 2.5 μm.

*Note:* This process can also be used to structure other metals (e.g.: copper, chromium, titanium), or combinations thereof.

23 When depositing metal combinations using sputtering, only a low sputtering power should be used to avoid spalling or cracking of the metals.
24 The PVA release layer allows rapid detachment of the membrane from its carrier and aids further processing.

## Method validation

**Fig. 2:**
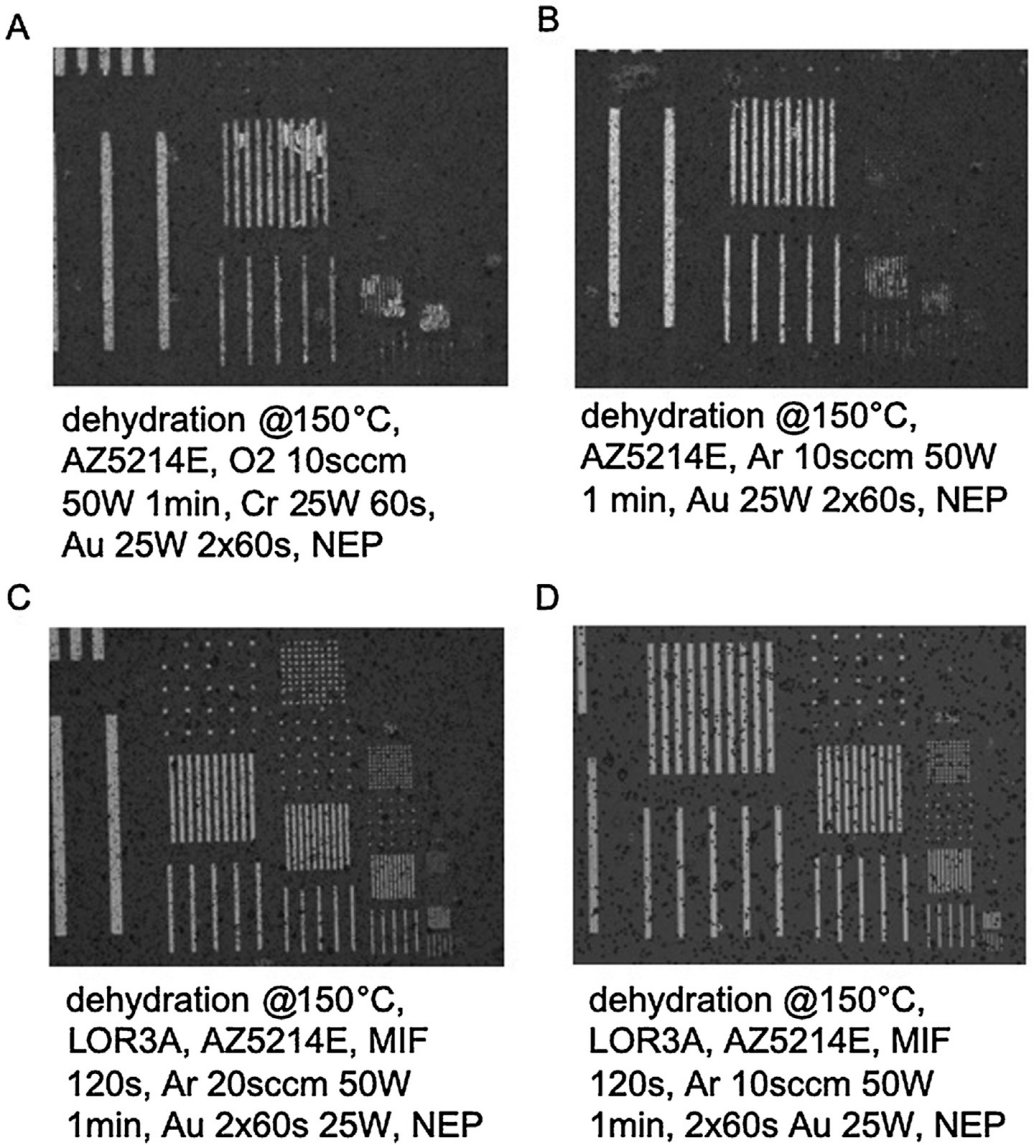
Microscopic images of test patterns fabricated with (A,B) single photoresist and (C,D) optimized dual-photoresist lift-off protocol. Black spots visible on the surface are 3 μm track-etched pores of the porous membrane.

**Fig. 3:**
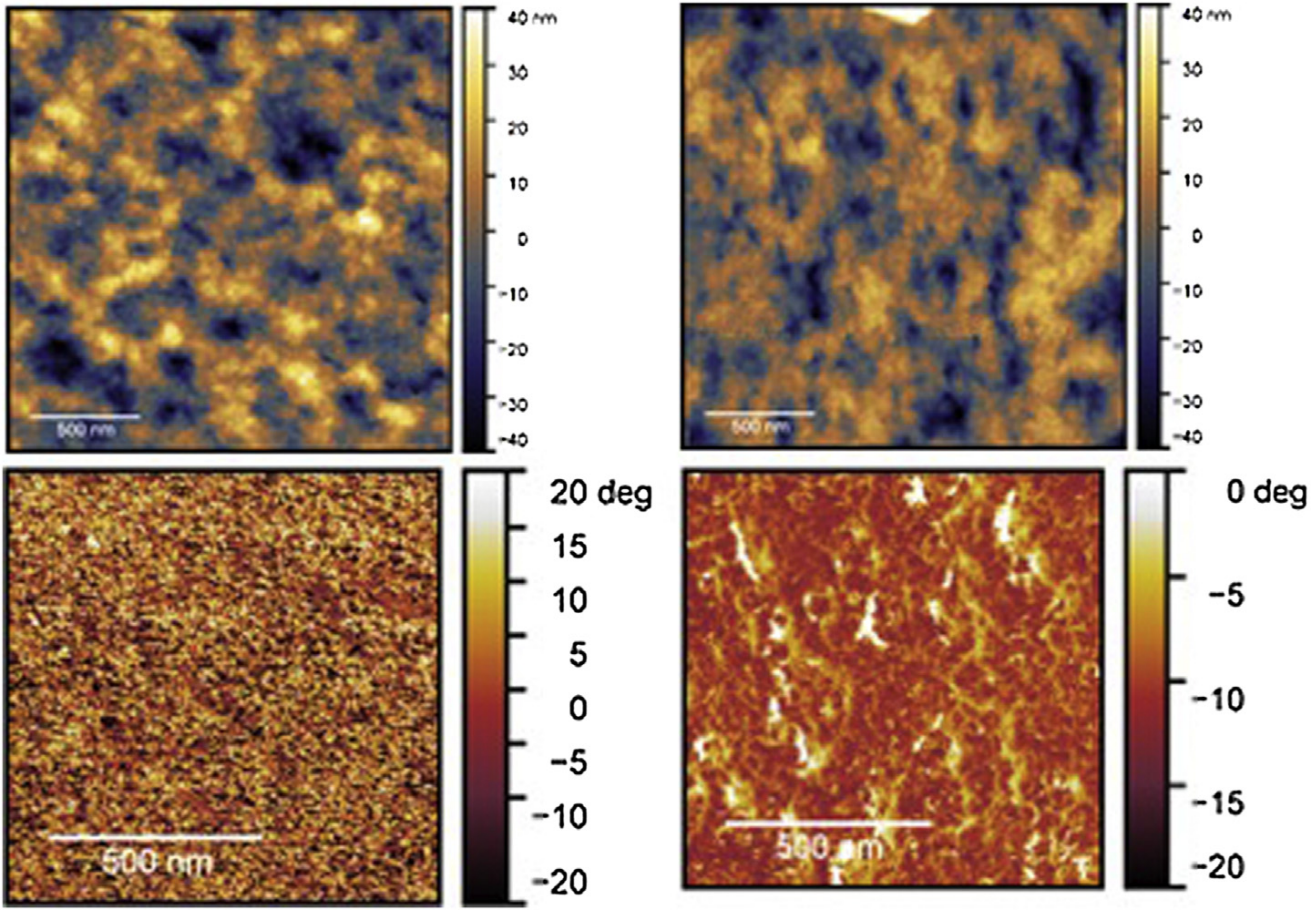
Atomic force micrographs of the height profile (top) and phase (bottom) of porous PET membranes before (left panel) and after (right panel) Ar-plasma at 10 sccm and 50 W. Scale bar, 500 nm.

Several process parameters such as plasma power, type, duration and etc. were constantly evaluated, resulting in the optimized protocol presented above. Overall process parameters and results are summarized in Table 1. The gold microstructures fabricated on porous membranes achieved a resolution down to 2.5 μm. Fig. 2 shows a comparison of four parameter sets, Fig. 2A and B show thin-film microelectrodes fabricated using single-layer resist lift-off. The PET substrate in Fig. 2A was subjected to an O_2_ plasma before Au deposition, while the substrate in Fig. 2B was subjected to an Ar plasma both with a power of 50 W and an oxygen flow rate of 10 sccm. Fig. 2C and D shows microelectrodes fabricated using a bi-layer resist fabrication approach, both substrates were subjected to Ar plasma with a power of 50 W. In case of the sample in Fig. 2C the Ar flow rate was 20 sccm while the flow rate in Fig. 2D was 10 sccm, resulting in better adhesion of Au to the substrate. Also, O_2_ plasma at 10 sccm and 50 W could achieve results comparable to the best Ar treated samples down to a resolution of 2.5 μm only in the presence of a 5 nm chromium adhesion layer. As shown also in Table 1, any fabrication approaches that used only a single photoresist were inferior in terms of resolution to the optimized bi-layer photoresist fabrication methods. Also evident is the influence of high plasma and metal deposition powers resulting in structural and functional artifacts including thin-film delamination or cracking/ spalling of the metal layer. The improved performance of the presented optimized bi-layer lift-off fabrication protocol is not caused by physical etching known to increase surface area. For instance, Fig. 3 shows that plasma-treated PET membranes display similar surface roughness, thus area, in comparison to pristine non-modified samples. Additional phase images however revealed an increase in hydrophilicity due to incorporation of more hydrophilic surface groups, which significantly improved metal film adhesion.

**Table 1:** Summary of the process optimization. +++ excellent, ++ good, + acceptable, - failed.

Paramter set	Plasma type	Plasma Power	Flow rate/ Pressure	Plasma time	Adhesion layer	Bilayer Photoresist	Deposition Power	Result
**1**	Ar	50 W	20 sccm	60 s	Cr	No	50 W	–
**2**	O_2_	100W	0,7 Torr	60 s	Cr	No	50 W	–
**3**	O_2_	100 W	0,7 Torr	120 s	Cr	No	50 W	–
**4**	O_2_	300 W	0,7 Torr	45s	Cr	No	50 W	–
**5**	Ar	10 W	10 sccm	60 s	Cr	No	25 W	–
**6**	Ar	10W	10 sccm	30 s	Cr	No	25 W	–
**7**	O_2_	10W	10 sccm	60 s	Cr	No	25 W	+
**8**	O_2_	50 W	10 sccm	60 s	Cr	No	25 W	+
**9**	Ar	100 W	10 sccm	60 s	Cr	No	25 W	+
**10**	O_2_	100W	10 sccm	30 s	Cr	No	25 W	+
**11**	Ar	50 W	10 sccm	60 s	None	LOR3A/AZ5214E	25 W	+++
**12**	Ar	50 W	20 sccm	60 s	None	LOR3A/AZ5214E	25 W	++
**13**	Ar	50 W	10 sccm	60 s	None	No	25 W	–
**14**	O_2_	50 W	10 sccm	60 s	Cr	No	25 W	–
**15**	O_2_	50 W	10 sccm	60 s	Cr	LOR3A/AZ5214E	25 W	+++

**Fig. 4:**
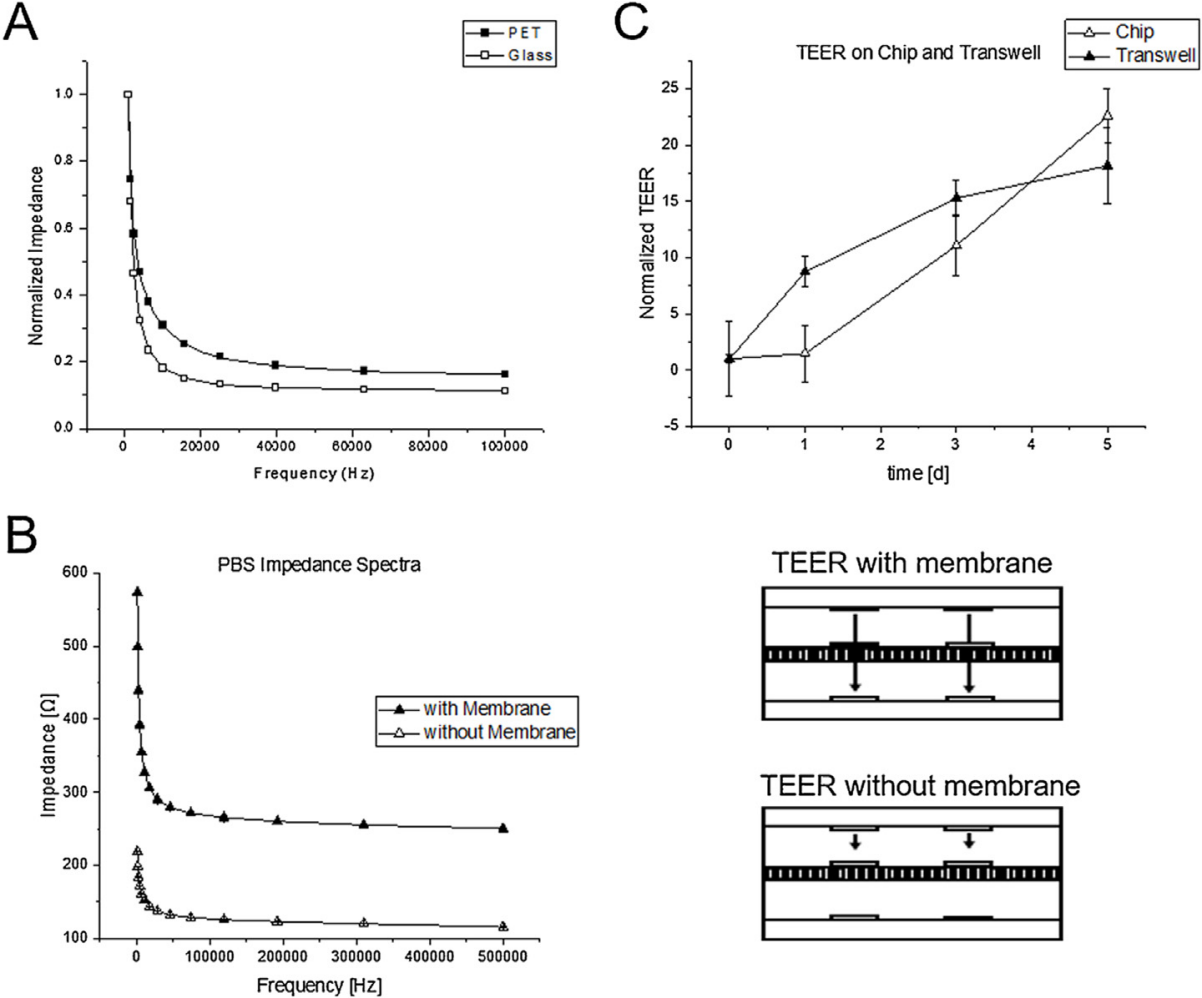
(A) Comparison of the frequency behavior of 75 nm gold thin-film electrodes fabricated in glass with conventional single photoresist lift-off (Glass) in comparison to thin-film electrodes fabricated on porous PET membranes using the optimized plasma-assisted protocol (PET). (B) Impedance spectroscopy of tetra-polar TEER measurements in with and without measuring the membrane resistance. (C) Comparison of TEER monitoring of Bewo epithelial cells seeded at 100k/cm^2^ over a time course of 5 days using membrane-bound electrodes (Chip) in comparison to EVOM2 read-out in 3 μm Transwells®. (n = 3).

To validate the electrode quality, frequency sweeps were recorded for Dulbecco’s Minimal Essential Medium (DMEM) on 80 nm gold thin-film electrodes either fabricated on glass or PET track-etched membranes. The electrodes were connected to a VMP-3 Multichannel potentiostat (Bio-Logic Science Instruments, France) using spring contacts. Impedimetric measurements were performed with an excitation voltage of 50 mV and a frequency between 1 Hz and 500 kHz. Fig. 4A confirms that the frequency behavior on PET membranes is similar to microelectrodes fabricated on glass, thus quality of the microfabricated electrodes is not affected by the optimized bi-layer lift-off protocol presented. Fig. 4B shows that the presented membrane-bound high-resolution thin-film electrodes can be used in a state-of-the-art tetrapolar measurement setup frequently used for electric resistance evaluation of cell-based barrier models (transepithelial/-endothelial resistance, TEER) to eliminate the artificial resistance of the porous membrane. This can improve the overall sensor performance because industrial track-etched membranes are known to have high batch-to-batch variations due to mass production of track-etched membranes. As a final application, trans-epithelial resistance of Bewo placental epithelial cells at a seeding density of 100k cells/ cm^2^ in DMEM supplemented with 10 % fetal calf serum and 1 % antibiotics mix was monitored using a tetrapolar TEER setup. Thin-film gold electrodes on 3 μm porous PET membranes were compared to conventional Transwell® 3 μm inserts tested with an EVOM2 Vohm meter equipped with an STX3 Ag/AgCl electrode. [3] Fig. 4C shows that membrane-bound thin-film electrodes can be readily used in any TEER or impedance-based measurement setup and can monitor cell barrier dynamics over several days.

In summary, we have optimized a metal deposition method for the fabrication of high-resolution microstructures down to 2.5 μm on porous PET membranes. This improved resolution in combination of using ultra-thin porous membranes enables the development of novel microfluidic devices in addition to other applications, which require conformal electrodes with excellent resolution and high surface adhesion.
